# The efficacy of intra-articular triamcinolone acetonide 10 mg vs. 40 mg in patients with knee osteoarthritis: a non-inferiority, randomized, controlled, double-blind, multicenter study

**DOI:** 10.1186/s12891-023-06191-6

**Published:** 2023-02-03

**Authors:** Komchan Utamawatin, Ong-art Phruetthiphat, Rit Apinyankul, Sumapa Chaiamnuay

**Affiliations:** 1 Department of Medicine, Phramongkutklao Hospital and College of Medicine, Bangkok, Thailand; 2 Department of Orthopedics, Phramongkutklao Hospital and College of Medicine, Bangkok, Thailand; 3 Department of Orthopaedics, Faculty of Medicine, Khon Kaen University, Khon Kaen, Thailand; 4 Rheumatic Disease Unit, Department of Internal Medicine, Phramongkutklao Hospital and Phramongkutklao College of Medicine, 315 Ratchawithi Road Ratchathewi District, Bangkok, 10400 Thailand

**Keywords:** Osteoarthritis, Intra-articular corticosteriod injection, Triamcinolone acetonide, Non-inferiority trial, Knee

## Abstract

**Background:**

Intra-articular (IA) corticosteroid injection is recommended in refractory knee osteoarthritis patients. However, 40-mg of triamcinolone IA every 3 months for 2 years reduces cartilage volume as compared to saline IA.

**Objective:**

To determine the non-inferiority of 10-mg versus 40-mg of triamcinolone acetonide (TA) for treatment of pain in symptomatic knee osteoarthritis at week 12.

**Methods:**

This was a double-blind, randomized, controlled trial conducted in 84 symptomatic knee osteoarthritis patients. The 10-mg or 40-mg of TA were 1:1 randomized and injected to the affected knees. The primary outcome was the 12-week difference from baseline in pain VAS, with a pre-specified lower margin for non-inferiority of 10 mm. The measuring instruments used were: Visual analog scale (VAS: 0–10), modified Western Ontario and McMaster Universities Osteoarthritis Index (WOMAC), EuroQol Group 5 Dimensions (EQ5D), Knee Injuries and Osteoarthritis Outcome Score (KOOS) questionnaire, chair standing test and 20-m walking time at baseline, at week 4, and week 12 after randomization. Adverse events were recorded.

**Results:**

Baseline characteristics were similar between two groups. The mean differences of pain VAS (95% confidence interval: CI) between the two groups at baseline and week 12 were 0.8 (-0.8, 2.4) with p of 0.002 for non-inferiority. There were no differences in pain reduction and quality of life improvement between 10-mg and 40-mg groups. The mean differences (95%CI) of WOMAC, KOOS pain, EQ5D and KOOS quality of life between baseline and week 12 were 0.4 (-1.1, 1.9). -8.7 (-21.3, 3.9), 1.3(-7.1, 9.6) and 1.8 (-11.5, 15.0), respectively.

There were significant improvements in pain and quality of life between baseline and week 12 in both groups.

**Conclusion:**

The 10 mg of TA is non-inferior to 40 mg TA in improving pain in patients with symptomatic knee OA. Both 10 mg and 40 mg of TA significantly improved pain and quality of life in patients with symptomatic knee OA.

**Trial registration:**

TCTR, I TCTR20210224002. Retrospectively registered 24 February 2021, http://www.thaiclinicaltrials.org/show/TCTR20210224002

## Introduction

The global prevalence of osteoarthritis (OA) has been increasing from 247.51 million in 1990 to 527.81 million in 2019 particularly at the knee and hip sites. OA of the knee contributed the most to the overall burden and has become the leading cause of disability [1]. Current managements of osteoarthritis include pharmacological and non-pharmacological management. There are several medications to relieve pain, but there is no proven medication to slow the progression of the disease. Pain control is the cornerstone of the OA treatment. The 2019 ESCEO guideline recommended using oral symptomatic slow acting drugs for osteoarthritis (SYSADOA) and acetaminophen as the first line pain reliever. Oral non-steroidal anti-inflammatory drugs (NSAIDs) are the second line drugs to be used with caution, because elderly patients usually have underlying diseases such as metabolic diseases, kidney diseases or cardiovascular diseases [2]. The 2019 Osteoarthritis Research Society International (OARSI) recommended using topical NSAIDs as the first line, followed by oral NSAIDs and did not recommend the use of SYSADOA [3].

Several studies found that intraarticular (IA) corticosteroids for example triamcinolone acetonide (TA), triamcinolone hexacetonide, methylprednisolone acetate and hydrocortisone improve symptoms in patients with osteoarthritis of the knee [4–6]. Therefore, it was recommended by most guidelines [2, 3, 7, 8] as the third line for pain control or for those with contraindication for other medications. However, there is no consensus for the proper dose and type of IA corticosteroid for the treatment of symptomatic knee OA. Several trials reported that 40 milligrams (mg) of triamcinolone intraarticular injection is effective in controlling pain in knee OA [9–13].

Although IA corticosteroids demonstrated efficacies in relieving pain in knee OA which allows patients to utilize the joints with minimal pain, there is an ongoing concern about the cartilage safety and long-term OA progression. Furthermore, the catabolic effects of IA corticosteroids might have negative effects to chondrocytes, which in turns results in long-term damage to the joints [6]. McAlindon et al. reported that patients who received the 40 mg of IA TA injection every 3 months for 2 years might have a greater cartilage volume loss than those who received saline intraarticular injection [9].

Given the proven efficacies of IA corticosteroid for knee OA and the concerns about long-term joint damage, the lower dose of IA corticosteroid could be effective in the treatment of knee OA. Intraarticular triamcinolone 10 mg has been effectively used for the treatment of knee OA in clinical practice. However, there has been no randomized controlled trial to demonstrate its efficacy. We performed a non-inferiority, multi-centered, randomized, double-blinded, controlled trial to compare between the effectiveness of IA TA 10 mg with IA TA40 mg for the treatment of osteoarthritis of the knee.

## Materials and methods

### Patients

Patients were recruited from outpatient clinics of rheumatology and orthopedics department at Phramongkutklao Hospital, Bangkok, and an outpatient clinic of orthopedics at Srinagarind Hospital, Khon Kaen, from 1 August 2019 to 28 February 2021.

Eligible patients were 50 years of age or older who were diagnosed with primary osteoarthritis of knee (either monoliteral or bilateral) according to the American College of Rheumatology (ACR) clinical classification criteria [14] with Kellgren-Lawrence radiographic severity grade 2–4 [14] with moderate to severe pain defined as pain visual analog scale (pVAS) more than or equal to 40 mm. Patients were intolerant or not responsive to non-steroidal anti-inflammatory drugs and indicated for IA corticosteroid injection according to the 2019 ACR Guideline for the Management of Osteoarthritis of the Hand, Hip, and Knee [7].

Exclusion criteria were patients who had contraindication for IA injection such as overlying skin and soft tissue infection, uncontrolled bleeding diathesis, had IA glucocorticoid injection in the past 3 months, had IA hyaluronic acid in the past 6 months, patients with secondary OA due to inflammatory arthritis, trauma or infection or history of knee replacement in the same joint and planning for knee surgery within 3 months.

### Study design

This was a non-inferiority, multi-centered, randomized, double-blinded, controlled trial. This study was approved by the Institutional Board Review of the Royal Thai Army Medical Department and Khon Kaen University (Approval number IRBRTA 700/2562 and HE621569) and was retrospectively registered at Thai clinical trials registry (TCTR20210224002) and approved on 24/02/2021. The study was conducted in accordance with the Declaration of Helsinki and followed the International Conference for Harmonization Guidelines for Good Clinical Practice. Written informed consent was obtained from each patient before study participation.

### Trial procedure: randomization and treatment allocation

Consecutive eligible patients in all sites who agreed to participate in the study were randomized by block of four by a study coordinator to receive intraarticular injection of either 10 mg of Triamcinolone acetonide (TA) (10 mg per milliliter, ml) or 40 mg (40 mg per ml) of TA. All TA were manufactured by L.B.S. Laboratory Limited. The IA TA injections were prepared by research nurses. Either 10 or 40 mg of TA was drawn into a syringe plus 3 ml of 1% lidocaine and then the syringe was taped to blind the investigators. The knee joint injections were performed under sterile condition by a certified rheumatologist (SC) or certified orthopedists (OP and RA) with the same technique (suprapatellar approach). The synovial fluid, if present, was maximally removed prior to the IA corticosteroid injection. Synovial fluid was analyzed for cell count and crystals.

### Outcome measures

Patients were assessed for the pain by visual analog scale (pVAS:0–10), global visual analog scale (gVAS:0–10), modified Thai version of the Western Ontario and McMaster osteoarthritis index (WOMAC) pain subscale range, 0 [no pain]-10 [extreme pain], the stiffness subscale range, 0 [no stiffness]-10 [extreme stiffness], the joint usage subscale range, 0 [excellent]-10 [unable] [15], Thai version of Knee and Osteoarthritis Outcome Score (KOOS) [16, 17], EQ-5D-5L [18], 20-m walk time and chair stand test at baseline, 4 weeks and 12 weeks after randomization. The primary outcome was the differences in changes in pVAS between baseline and week 12 between the two treatment groups.

Acetaminophen at the maximum dose of 3 g per day with a 12-h washout before each follow-up visit was offered as a rescue for pain. The numbers of a 500 mg of acetaminophen tablet used were recorded at each visit. NSAIDs and oral glucocorticoid were not allowed during the study. Adverse events were recorded from screening to the end of the study. Injection site pain, acute pseudo-septic or septic arthritis and bleeding were recorded at the baseline visit.

During the COVID-19 pandemic, if patients were not able to come to the hospital, questionnaires were sent to patients and returned to the researchers by postal or electronic mails. At baseline visit, patients and their caregivers were instructed by the research assistant and a standard short video how to perform a chair stand test and record a 20-m walk time.

### Statistical analysis

The sample size was calculated based on the primary outcome (the differences in the change in pVAS between randomization and week 12). From the meta-analysis by Juni P, et al., [5], the mean differences in pVAS before and after intraarticular injection were -2.8 cm and -1.8 cm with the standard deviation (SD) of 1.84 in the glucocorticoid and placebo groups, respectively. From the previous randomized controlled study which examined efficacy of rofecoxib, ibuprofen and placebo, the minimal perceptible clinical improvement for WOMAC pain was about 10 mm from the 100 mm normalized VAS [19]. Therefore, the non-inferiority margin was set as 10 out of 100 mm for pVAS. A type I error risk was set at 5% with an 80% power, which gave the sample size as 42 patients for each group. The statistical analyses were performed by pre-defined intention-to-treat (ITT) and per protocol (PP) analyses.

Baseline characteristics were analyzed using descriptive statistics. Categorical data are presented as number and percentage. Continuous variables are presented as mean and SD. The differences in pain VAS, global VAS, WOMAC score, KOOS score and EQ5D between baseline, fourth week and twelfth week were compared by independent t test, Chi-square test and generalized estimating equation (GEE). All statistical analyses were performed by using STATA 17. Statistical significance was defined as *p*-value < 0.05.

## Results

**Fig. 1:**
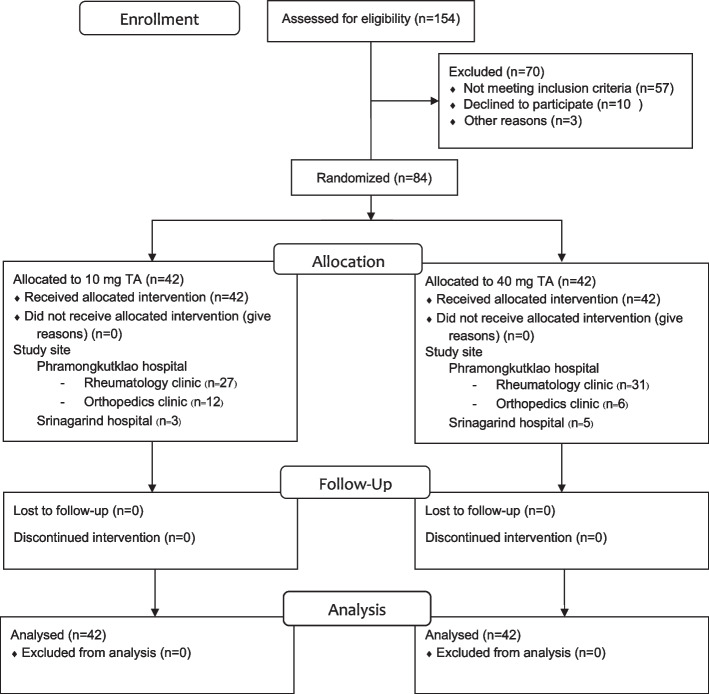
Flowchart of patients with symptomatic knee osteoarthritis throughout the study

Eighty-four patients were enrolled in this study and the CONSORT diagram was shown in Fig. 1. One hundred and fifty-four patients were screened. Seventy patients met the exclusion criteria. Six patients declined to participate. Eighty-four patients were enrolled and randomized into two groups (42 patients in each group) to receive IA TA 10 mg or 40 mg. All patients completed baseline, week 4 and week 12 visits. Thus, the ITT and PP analyzed were similar. Baseline demographic and clinical characteristics were similar between two groups. Radiographic severity was similar between two groups.

Baseline characteristics of all participants are summarized in Table 1. Most were women (66%), moderately overweight (body mass index 26.4 ± 2.9 kg/m2) with a mean (SD) age of 66.9 (10.2) years old. About 45% of patients had knee OA severity grade IV according to the modified Kellgren-Lawrence radiographic scoring. Knee OA was unilateral in half of the patients. Most of them had a uni-compartmental tibiofemoral OA (67.5%) without associated patellofemoral pain syndrome (75.0%).

**Table 1:** Baseline characteristics of the study participants

Triamcinolone acetonide	10 mg (*n* = 42)	40 mg (*n* = 42)	*p*-value
Age, mean ± SD^a^	**69.6 ± 9.2**	**69.9 ± 11.3**	**0.9**
Female, n (%)	**35 (83)**	**35 (83)**	**1.0**
Body mass index, mean ± SD^a^	**24.9 ± 3.5**	**25.4 ± 3.9**	**0.5**
Comorbidity, n (%)			
Hypertension	**25 (60)**	**28 (67)**	**0.5**
Diabetics mellitus	**10 (24)**	**13 (31)**	**0.5**
Dyslipidemia	**29 (69)**	**23 (55)**	**0.2**
Kellgren and Lawrence grade, n (%)			**0.7**
Grade 2	**11 (26)**	**9 (21)**	
Grade 3	**11 (26)**	**15 (36)**	
Grade 4	**20 (48)**	**18 (43)**	
Joint effusion, n (%)	**12 (28)**	**15 (36)**	**0.4**

^a^Standard deviation

There were no differences in baseline osteoarthritis pain, disease severity, quality of life and function as measured by pVAS, gVAS, modified Thai version of WOMAC, Thai version of KOOS, EQ-5D-5L, 20-m walk time and chair stand between two groups. Those data were summarized in Table 2.

**Table 2:** Baseline assessments of the study participants

**Triamcinolone acetonide**	**10 mg** **(*n* = 42)**	**40 mg** **(*n* = 42)**	***p*-value**
**Pain VAS, Mean ± SD**	**5.2 ± 2.4**	**4.9 ± 2.7**	**0.6**
**Global VAS, Mean ± SD**	**4.0 ± 2.8**	**4.0 ± 3.1**	**0.9**
**Thai WOMAC score, Mean ± SD**			
**Pain**	**4.9 ± 2.1**	**4.7 ± 2.5**	**0.7**
**Stiffness**	**3.4 ± 2.9**	**3.0 ± 2.7**	**0.4**
**Use**	**3.7 ± 2.5**	**3.7 ± 2.4**	**0.9**
**Total**	**4.0 ± 2.1**	**3.8 ± 2.2**	**0.6**
**EQ5D, Mean ± SD**			
**Utility**	**0.8 ± 0.2**	**0.8 ± 0.2**	**0.7**
**Health**	**73.6 ± 14.4**	**70.2 ± 15.5**	**0.3**
**Thai KOOS, Mean ± SD**			
**Pain**	**54.1 ± 17.5**	**60.8 ± 19.3**	**0.1**
**Symptom**	**58.4 ± 19.3**	**64.5 ± 19.4**	**0.2**
**Activities of daily living**	**56.3 ± 19.4**	**61.6 ± 20.2**	**0.2**
**Sports/recreation**	**23.0 ± 21.2**	**28.8 ± 23.0**	**0.3**
**Quality of life**	**35.3 ± 17.6**	**35.2 ± 16.4**	**1.0**
**Chair stand test, Mean ± SD**	**6.2 ± 4.3**	**6.5 ± 3.6**	**0.7**
**20-m walk, Mean ± SD**	**53.5 ± 55.5**	**41.9 ± 48.1**	**0.3**

*SD* = Standard deviation,* VAS* = Visual analog scale,* EQ5D* = EuroQol-5 dimension,* KOOS* = The Knee injury and osteoarthritis outcome score *WOMAC* = The Western ontario and mcMaster universities osteoarthritis Index

### Primary outcome (pain visual analog scale)

**Fig. 2:**
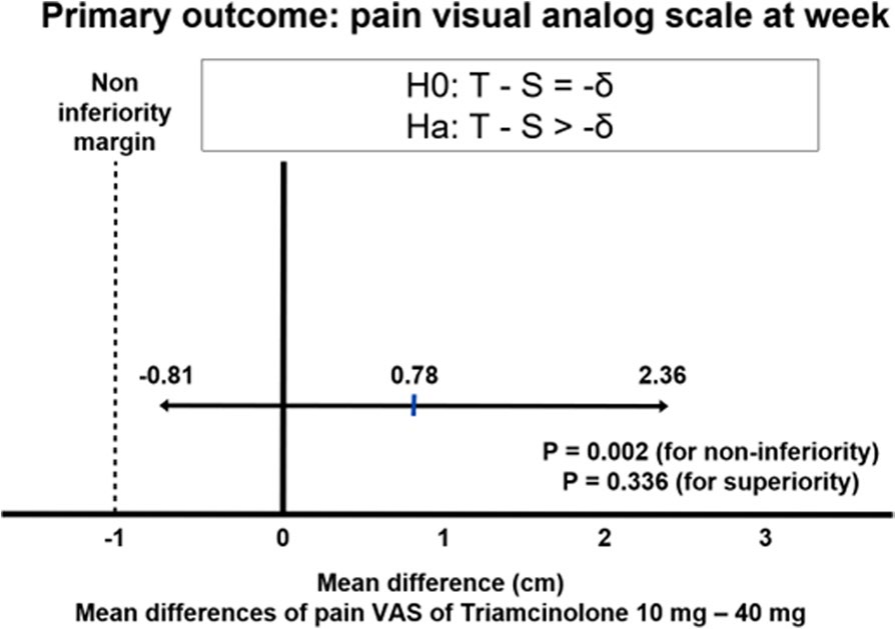
Non-inferiority of intraarticular Triamcinolone 10 mg versus Triamcinolone 40 mg for the treatment of symptomatic knee osteoarthritis

As shown in Fig. 2 the mean difference (95% CI) of pVAS between 10 and 40 mg group at week 12 and baseline was 0.8 (-0.8, 2.4) with p-value of 0.002 for non-inferiority and 0.34 for superiority. The 10 mg IA TA injection is non-inferior to 40 mg IA TA injection for the treatment of painful knee osteoarthritis.

Both 10 and 40 mg TA significantly improved pVAS at week 12 compared to baseline. The mean differences (95%CI) in pVAS scores at week 12 and baseline were -2.2 (-2.3, -2.1) and -1.4 (-1.5, -1.3) in 10 mg and 40 mg groups, respectively and the *p*-values were < 0.001 in both groups.

### Secondary outcomes

The efficacy data of 10 and 40 mg TA intraarticular were reported in detail in Table 3. The significant improvement in pain was observed at weeks 4 and 12 in both groups. Mean (95%CI) differences of pain domain in WOMAC and KOOS at baseline and at week 12 in 10 mg TA group were -1.2 (-1.3, -1.1) and 12.8 (11.9, 13.7), respectively and in 40 mg TA group were -0.8 (-0.9, -0.6), 4.1 (2.7, 5.4), respectively.

**Table 3:** Efficacy of triamcinolone acetonide 10 mg and 40 mg intraarticular at 4 and Week 12 s after the injection (intention to treat and per protocol analyses)

	**10 mg (*n* = 42)**			**40 mg (*n* = 42)**			**Mean difference (95%CI)**	***p*-value**
	**Mean ± SD**	**Mean change (95%CI)**	***p***	**Mean ± SD**	**Mean change (95%CI)**	***p***		
**Visual analog scale**								
**Pain**								
Baseline	5.2 ± 2.4	Reference	1	4.9 ± 2.7	Reference	1	Reference	1
Week 4	3.2 ± 2.2	-2.0 (-2.1, -1.9)	< 0.01	3.3 ± 2.5	-1.5 (-1.6, -1.4)	< 0.001	0.5 (-1.1, 2.1)	0.54
Week 12	3.0 ± 2.7	-2.2 (-2.3, -2.1)	< 0.01	3.5 ± 3.2	-1.4 (-1.5, -1.3)	< 0.001	0.8 (-0.8, 2.4)	0.34
**Global**								
Baseline	4.0 ± 2.8	Reference	1	4.0 ± 3.1	Reference	1	Reference	1
Week 4	2.7 ± 2.3	-1.4 (-1.5, -1.3)	< 0.01	2.7 ± 2.8	-1.2 (-1.4, -1.1)	< 0.001	0.2 (-1.5, 1.8)	0.86
Week 12	2.6 ± 2.5	-1.5 (-1.6, -1.4)	< 0.01	2.8 ± 3.0	-1.2 (-1.3, -1.0)	< 0.001	0.3 (-1.4, 2.0)	0.71
**EQ5D: Utility**								
**Utility**								
Baseline	0.8 ± 0.2	Reference	1	0.8 ± 0.2	Reference	1	Reference	1
Week 4	0.9 ± 0.1	0.1 (0.1, 0.1)	< 0.01	0.8 ± 0.2	0.1 (0.1, 0.1)	< 0.001	-0.1 (-0.2, 0.1)	0.40
Week 12	0.9 ± 0.2	0.1 (0.1, 0.1)	< 0.01	0.8 ± 0.3	0.1 (0.0, 0.1)	< 0.001	-0.1 (-0.2, 0.0)	0.23
**The Thai Western Ontario and McMaster Universities Osteoarthritis Index Thai (WOMAC)**								
**Pain score (0–10)**								
Baseline	4.9 ± 2.1	Reference	1	4.7 ± 2.5	Reference	1	Reference	1
Week 4	3.8 ± 2.3	-1.1 (-1.2, -1.0)	< 0.01	3.8 ± 2.4	-0.9 (-1.0, -0.8)	< 0.01	0.2 (-1.3, 1.6)	0.84
Week 12	3.7 ± 2.5	-1.2 (-1.3, -1.1)	< 0.01	3.9 ± 2.9	-0.8 (-0.9, -0.6)	< 0.01	0.4 (-1.1, 1.9)	0.59
**Stiffness score (0–10)**								
Baseline	3.4 ± 2.9	Reference	1	3.0 ± 2.7	Reference	1	Reference	1
Week 4	2.3 ± 2.5	-1.1 (-1.2, -1.0)	< 0.01	2.3 ± 2.9	-0.6 (-0.8, -0.4)	< 0.01	0.5 (-1.2, 2.1)	0.57
Week 12	2.2 ± 2.3	-1.3 (-1.4, -1.2)	< 0.01	2.6 ± 3.0	-0.4 (-0.5, -0.3)	< 0.01	0.9 (-0.8, 2.5)	0.29
**Joint usage score (0–10)**								
Baseline	3.7 ± 2.5	Reference	1	3.7 ± 2.4	Reference	1	Reference	1
Week 4	2.6 ± 2.4	-1.1 (-1.3, -1.0)	< 0.01	2.7 ± 2.6	-1.0 (-1.1, -0.9)	< 0.01	0.1 (-1.4, 1.6)	0.90
Week 12	2.6 ± 2.5	-1.2 (-1.3, -1.0)	< 0.01	2.8 ± 2.9	-0.9 (-1.0, -0.7)	< 0.01	0.3 (-1.2, 1.8)	0.70
**Total score (0–10)**								
Baseline	4.0 ± 2.1	Reference	1	3.8 ± 2.2	Reference	1	Reference	1
Week 4	2.9 ± 2.0	-1.1 (-1.2, -1.0)	< 0.01	2.9 ± 2.3	-0.8 (-1.0, -0.7)	< 0.01	0.3 (-1.1, 1.6)	0.70
Week 12	2.8 ± 2.2	-1.2 (-1.3, -1.1)	< 0.01	3.1 ± 2.7	-0.7 (-0.8, -0.6)	< 0.01	0.5 (-0.8, 1.9)	0.45
**The Thai Knee injury and Osteoarthritis Outcome Score (Thai KOOS)**								
**Pain**								
Baseline	54.1 ± 17.5	Reference	1	60.8 ± 19.3	Reference	1	Reference	1
Week 4	65.6 ± 21.1	11.4 (10.6, 12.3)	< 0.01	68.79 ± 19.42	8.03 (6.68, 9.38)	< 0.01	-3.41 (-16.0, 9.2)	0.60
Week 12	66.9 ± 22.0	12.8 (11.9, 13.6)	< 0.01	64.81 ± 24.66	4.05 (2.7, 5.4)	< 0.01	-8.7 (-21.3, 3.9)	0.18
**Symptoms**								
Baseline	58.4 ± 19.3	Reference	1	64.5 ± 19.4	Reference	1	Reference	1
Week 4	69.9 ± 19.2	11.4 (10.6, 12.3)	< 0.01	74.1 ± 16.3	9.6 (8.8, 10.4)	< 0.01	-1.8 (-13.4, 9.8)	0.76
Week 12	71.6 ± 20.0	13.2 (12.3, 14.0)	< 0.01	69.9 ± 20.7	5.4 (4.6, 6.2)	< 0.01	-7.8 (-19.4, 3.9)	0.19
**Activities daily living function**								
Baseline	56.3 ± 19.4	Reference	1	61.6 ± 20.2	Reference	1	Reference	1
Week 4	64.7 ± 23.3	8.4 (7.4, 9.3)	< 0.01	65.8 ± 20.4	4.1 (2.8, 5.5)	< 0.01	-4.3 (-17.5, 9.0)	0.53
Week 12	62.8 ± 24.0	6.5 (5.6, 7.5)	< 0.01	65.0 ± 23.2	3.3 (2.0, 4.7)	< 0.01	-3.2 (-16.4, 10.1)	0.64
**Sport and recreation function**								
Baseline	23.0 ± 21.2	Reference	1	28.8 ± 23.0	Reference	1	Reference	1
Week 4	31.7 ± 29.2	8.7 (6.1, 11.3)	< 0.01	32.1 ± 23.3	3.4 (1.8, 4.9)	< 0.01	-5.3 (-20.9, 10.3)	0.50
Week 12	32.9 ± 29.0	9.9 (7.3, 12.5)	< 0.01	29.4 ± 25.8	0.6 (-0.9, 2.1)	0.44	-9.3 (-24.9, 6.3)	0.24
**Quality of life**								
Baseline	35.3 ± 17.6	Reference	1	35.2 ± 16.4	Reference	1	Reference	1
Week 4	43.0 ± 24.1	7.6 (5.5, 9.7)	< 0.01	45.8 ± 20.1	10.6 (9.2, 12.0)	< 0.01	3.0 (-10.3, 16.3)	0.66
Week 12	47.8 ± 23.0	12.5 (10.4, 14.6)	< 0.01	49.4 ± 26.7	14.2 (12.9, 15.6)	< 0.01	1.8 (-11.5, 15.0)	0.80
**Sit up and time for 20 m walked**								
**Sit up**								
Baseline	6.2 ± 4.3	Reference	1	6.5 ± 3.6	Reference	1	Reference	1
Week 4	6.2 ± 4.4	0 (-0.2, 0.2)	1	6.6 ± 4.1	0.1 (-0.1, 0.3)	0.29	0.1 (-2.5, 2.7)	0.94
Week 12	6.3 ± 4.5	0.1 (-0.0, 0.3)	0.10	6.6 ± 4.7	0.0 (-0.3, 0.4)	0.88	-0.1 (-2.7, 2.5)	0.93
**Time for 20 m walked**								
Baseline	53.5 ± 55.5	Reference	1	41.9 ± 48.1	Reference	1	Reference	1
Week 4	55.5 ± 63.0	2.0 (0.2, 3.7)	0.03	35.9 ± 24.0	-6.0 (-8.2, -3.8)	< 0.01	-7.9 (-37.3, 21.4)	0.60
Week 12	53.9 ± 57.5	0.4 (-1.4, 2.1)	0.68	35.0 ± 23.4	-6.8 (-10.1, -3.5)	< 0.01	-7.2 (-36.7, 22.4)	0.63

*SD* for Standard deviation,* CI* for Confidence interval

The mean (95%CI) differences in gVAS scores at baseline and week 12 were -1.5 (-1.6, -1.4) and -1.2 (-1.3, -1.0) in 10 mg TA group and 40 mg TA group respectively. The mean (95%CI) difference in total Thai WOMAC score at baseline and week 12 were -1.2 (-1.3, -1.1) and -0.7 (-0.8, -0.6) in 10 mg TA group and 40 mg TA group respectively.

There were significant improvements in quality of life between baseline and at week 4 and 12 in both groups. Mean (95%CI) differences of EQ5D and KOOS quality of life at baseline and at week 12 in 10 mg TA group were 4.7 (3.9, 5.5), 12.5 (10.4, 14.6) and in 40 mg TA group were 5.9 (4.6, 7.3), 14.2 (12.9, 15.6).

The mean (SD) differences in Thai KOOS scores at baseline and week 12 in both groups were similar in all outcome domain assessments.

**Fig. 3:**
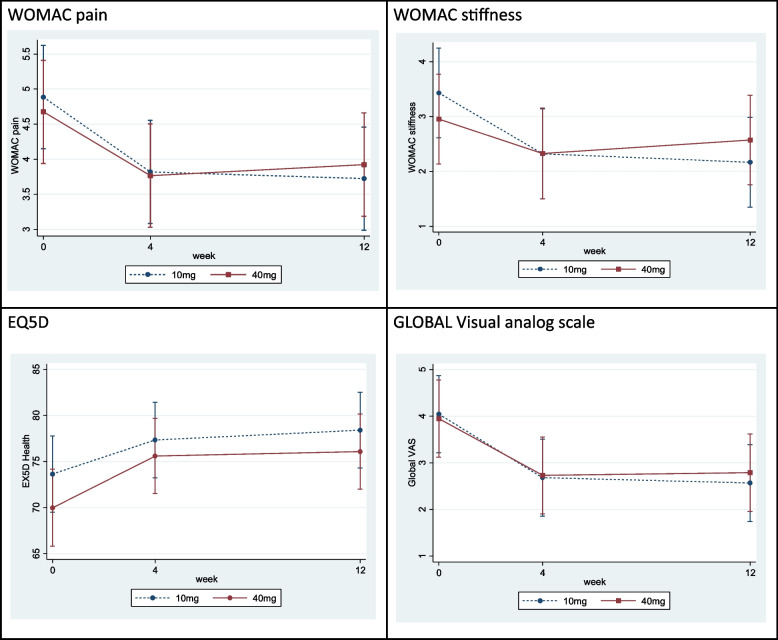
Treatment effects of triamcinolone acetonide 10 and 40 mg on secondary efficacy outcomes including WOMAC pain subscale (**a**), WOMAC stiffness subscale (**b**), EQ5D assessment (**c**) and global visual analog scale assessment (**d**)

The efficacies of TA 10 and 40 mg intraarticular as measured by WOMAC pain subscale, WOMAC stiffness subscale, EQ5D and gVAS are shown in Fig. 3.

There were no differences between two groups in the changes of functional assessment measured by the time to 20-m walk and the chair standing test between baseline and at week 4 and week 12. Patients in a 40 mg group walked faster at week 12 as compared to baseline with the mean difference of -6.8 (-10.1, -3.5) seconds. However, there was no difference in the time to 20-m walk between at baseline and at week 12 in the 10 mg group with the mean difference of the time to 20-m walk at week 12 compared with baseline was 0.4 (-1.4, 2.1) seconds. There were no differences in the chair stand test at baseline and at week 12 within and between groups.

The proportion of patients who need acetaminophen as a rescue therapy were similar between two groups (24% in 10 mg TA group, and 33% in 40 mg TA group; p-value 0.33) with the average use of 14 tablets per 12 weeks for both groups. No serious adverse events such as infection, severe bleeding or pseudo-septic reaction were reported during the study.

## Discussion

This study was the first randomized controlled trial which compared the efficacy of IA 10 mg and 40 mg TA injection for the treatment of symptomatic knee osteoarthritis. This trial reported the non-inferiority of the 10 mg TA compared with the 40 mg TA to reduce pain in patients with knee osteoarthritis. Both 10 mg and 40 mg TA were effective to relieve pain, improve function, quality of life and global assessment in patients with symptomatic knee osteoarthritis.

Patients in this study represent severe symptomatic knee osteoarthritis patients in a real-life practice who are indicated to receive IA glucocorticoid. The majority of patients are elderly, female and overweight. However, patients in this study are Asian which have lower body mass index compared to previous trials conducted in the Western countries [9]. Patients in this study are in the late stage of knee OA since about half of them had Kellgren-Lawrence radiographic severity grade 4 and about one-third of them had joint effusion. The baseline pain VAS and WOMAC pain subscales are comparable to other knee osteoarthritis trials [5, 9, 10, 20].

There was no published recommendation on the intraarticular dosages and type of glucocorticoid to treat symptomatic knee OA. Various types and dosages of IA glucocorticoids were previously investigated to treat symptomatic knee OA such as TA, triamcinolone hexatonide, methylprednisolone, prednisolone acetate, dexamethasone phosphate, and hydrocortisone. The most investigated IA glucocorticoid for the treatment of symptomatic knee OA is triamcinolone. It is still unclear whether the efficacy of intraarticular glucocorticoid for the treatment of symptomatic knee OA is dose dependent. The dose of 40 mg was examined in many previous studies and shown to be more effective to reduce pain as compared to normal saline intraarticular [5, 6, 9, 10]. Popma et al. conducted a 12-week randomized controlled trial in 2015, and compared the efficacy of 40 mg TA with a higher dose (80 mg TA) for the treatment of symptomatic knee OA patients [21]. He reported that a higher dose of TA had no additional benefit. The lowest dose of intraarticular triamcinolone examined to treat symptomatic knee OA is 20 mg of triamcinolone hexatonide. However, the benefits of 20 mg triamcinolone hexatonide intraarticular over placebo was evidenced only at weeks 1–2, but not at weeks 4–6 of follow-up. The authors concluded that 20 mg of triamcinolone hexatonide provided only short-term pain relief for knee OA [22, 23]. Another study examined different doses of rimexolone intraarticular which reported that rimexolone 20 and 40 mg intraarticular were superior to placebo to improve all clinical variables, while rimexolone 10 mg intraarticular was only improved joint tenderness [24].

From the real-world data, a survey among members of the ACR reported that the most used glucocorticoids for intraarticular, bursa and tendon sheath injection for diverse indications including osteoarthritis are triamcinolone and methylprednisolone, and the dose of 40 mg is the most used [25]. Therefore, 40 mg TA intraarticular injection was selected as the standard treatment in this study. However, 40 mg of TA intraarticular injection every 3 months for 2 years can decrease cartilage volume, which in turns, cause progression of knee OA [9].

The data from the Cochrane meta-analyses reported the benefit of IA glucocorticoid for symptomatic knee OA peaks at 2–4 weeks and wanes overtime with the moderate benefits after 1 to 2 weeks, small to moderate after 4 to 6 weeks, small after 13 weeks, and no evidence of any benefits at 26 weeks [5]. However, to prove the non-inferiority of the lower (10 mg) dose of triamcinolone to the higher dose (40 mg), we chose a 12-week follow-up time as the primary endpoint since at the shorter follow-up time (1–6 weeks), both doses might be similarly effective and longer follow-up time might be more appropriate to determine the difference in the efficacy between the two doses.

This study assessed various outcomes in symptomatic knee OA including pain, quality of life, and function. A 10 mg IA TA was not inferior to a 40 mg in improving pVAS in patients with symptomatic knee OA. There were no differences in other secondary outcomes. However, patients in a 40 mg TA group, not in a 10 mg group, walked faster at week 12 compared to baseline. However, there was no difference between the two groups.

There are a few limitations in this study. First, due to COVID-19 pandemic, some patients could not come to the hospital at a follow-up visit. However, the coordinator informed all patients and their family about how to perform a chair stand test and record a time to 20-m walk. The questionnaires were sent and returned electronically. Secondly, the long-term cartilage volume loss with 10 mg TA intraarticular was not examined, therefore, we cannot conclude that a 10 mg TA has less damage to the cartilage compared to a 40 mg TA. A further study with cartilage volume as a primary endpoint is warranted. Thirdly, this study had no placebo, thus, it could be inconclusive whether the efficacy of TA is from the TA itself or placebo effect. Fourthly, this study was conducted in only two centers in the same country.

## Conclusion

A 10 mg of TA IA injection provided non-inferior pain relief for symptomatic knee OA, as compared to a 40 mg of TA IA injection. The 10 mg of TA injection could be a safer option to treat symptomatic knee OA than a commonly used 40 mg of TA intraarticular. Both 10 and 40 mg TA intraarticular are effective to reduce pain and improve quality of life of patients with symptomatic knee OA.

## Data Availability

The data that support the findings of this study are available upon request to the correspondent author.
